# Gamma-Ray Analysis of Reed Samples from the Danube Delta

**DOI:** 10.3390/s25113257

**Published:** 2025-05-22

**Authors:** Ana Bianca Pavel, Sorin Ujeniuc, Gabriel Iordache, Irina Catianis, Catalina Gavrila, Albert Scrieciu, Radu Seremet, Iulian Andreicovici, Silvia Ise, Rares Suvaila

**Affiliations:** 1 NIRD GeoEcoMar, 030167 Bucharest, Romania; biancapavel@geoecomar.ro (A.B.P.); gabriel.iordache@geoecomar.ro (G.I.); irina.catianis@geoecomar.ro (I.C.); catalina.gavrila@geoecomar.ro (C.G.); albert.scrieciu@geoecomar.ro (A.S.); 2 Horia Hulubei NIRD IFIN-HH, 077125 Bucharest, Romania; ujeniuc.sorin@nipne.ro (S.U.); iulian-florin.andreicovici@cern.ch (I.A.); silvia.ise@nipne.ro (S.I.); 3 Faculty of Physics, University of Bucharest, 030018 Bucharest, Romania; r.seremet@cpce.net

**Keywords:** environmental radioactivity, Danube Delta, reed samples, pattern analysis

## Abstract

Gamma-ray analysis is a widely used technique for radioactive element characterization in environmental samples, contributing significantly to natural and anthropogenic radioactivity evaluations, particularly in areas such as natural reservations or regions that have been affected by nuclear pollutants. As the Danube Delta belongs to both categories, we decided to conduct a study in order to find out whether gamma spectroscopy is suited for pattern identification in common biota constituents such as reed and whether anthropogenic tracers can still be found in the samples. The answer to both questions is affirmative, as shown by the pattern and cluster analyses. Furthermore, our conclusions point out that it would be interesting to extend the spectroscopy and correlation studies to sediment and trophic chains over a certain period in order to obtain the transfer factors and information on radionuclide dynamics. The HPGe detector used proves this is the best class of sensing devices for such purposes.

## 1. Introduction

Numerous studies involving elemental analysis of biological samples have been performed over the last years in the Danube Delta, which is the largest well-preserved Delta in Europe. However, gamma-ray analysis of such samples lacks information on large areas, which is not only deficient for the elemental analysis itself but also a missing link in the sediment-to-trophic levels relationship study. In general, the geological influence of background radiation is complex [1], and a dose assessment is a complex procedure that depends on a multitude of factors [2].

The evaluation of the radionuclide activity in the environment, including marine and lacustrine areas, for purposes ranging from cause analysis to transfer factor estimation, is a broad area of research [3,4,5,6,7,8,9,10,11], involving laboratory activities but sometimes also consistent in situ capabilities, as described in [12]. Before engaging in any in situ campaign, in situations like the present one, it is necessary to find out whether the laboratory protocol is good enough for qualitative and quantitative determinations. For this purpose, we sampled common reed from different locations within the Danube Delta in order to find out whether radionuclides from the natural series are clearly displayed in the spectra, given the available shield and detection system, within a reasonable time interval (about a one-day acquisition per sample). Further, we ought to build a database of reference materials for the Danube River, with an emphasis on this area, from biota components to sediment [13], the latter having, a priori, the lowest variance for a given area. If the radionuclides (series, non-series, and anthropogenic) are clearly determined, the next step in the analysis process is to search for correlations, as the latter would help us build a valid transfer model in order to obtain as much information as possible with a minimum of measurements (which of course cannot be lower than a few thousands).

**Figure 1:**
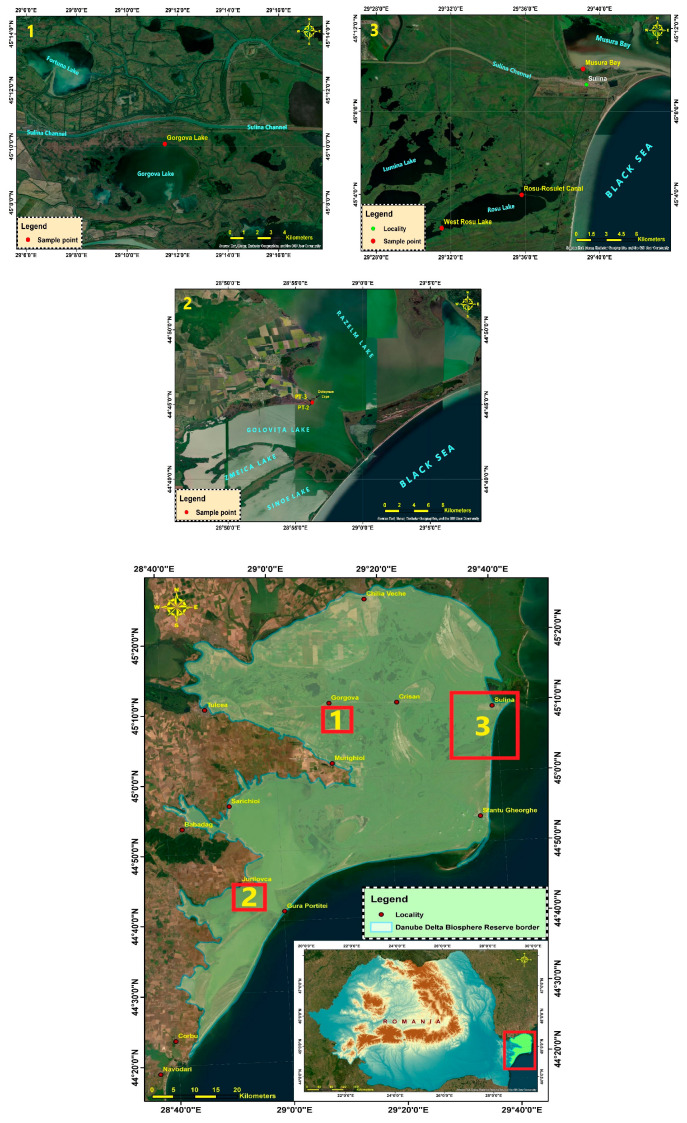
Sampling sites within the Danube Delta. 1—Gorgova lake Site; 2—Razelm Sinoe lagoon Complex; 3—Rosu-Rosulet Complex lakes (Source: Esri, Earth star Geographics, and the GIS User Community).

After collecting samples from the areas presented in Figure 1, as well as preparing and measuring them as described in the next section, we concluded that a gamma-ray analysis is the best available non-destructive method for such samples (as the prepared samples are kept intact and can be further analyzed following other protocols), and studies in this respect open the way for robust environmental characterization and powerful covariance studies.

HPGe detectors are clearly the most adapted sensors for collecting such spectra, as both their high efficiency and energy resolution qualify them in this regard. Here, an *n*-type detector was used, as this type of semiconductor also enables the detection of low-energy quanta (down to some 5 keV), with good efficiency due to their micrometric outer layer and entrance window. This class of detectors has been continuously developed for multiple applications, along with shielding devices, electronics, and associated software, with great success in environmental applications. The results of this study prove the superiority of HPGe detectors, even in a test setup, compared, for example, to NaI ones.

An additional sample analysis after neutron irradiation was also performed in order to extend the detection capability toward induced gamma emissions by nuclei, which are not radioactive or in an excited state at the sampling moment, but this is not part of the non-destructive protocol, because a neutron-activated sample is considered destroyed.

## 2. Materials and Methods

### 2.1. Study Area

The Lumina–Roșu plain is located in the Southern Danube Delta area (Sulina–Sf. Gheorghe), on the marine side of the delta plain. This lowland is a very vast one, situated between the Caraorman grind (west), Sulina Branch (north), the Black Sea shore (east), and St. George Branch (south). The hollow includes three big lakes—Rosu (1445 ha.), Lumina (1367 ha.), and Puiu (865 ha.), and many smaller lakes, among which the most important are Iacub, Roșuleț, Puiuleț, Vătafu, Lungu, Porcu, Rotund, Macuhova, Potcoava, and Erenciuc [14,15].

The Gorgova–Uzlina plain is located in the fluvial area of the delta, between Sulina Branch, St. Gheorge Branch, and the Caraorman grind, and it is characterized by a hydrographical network that is influenced by the two mentioned branches, the Litcov channel (built between 1930 and 1940) and the Perivolovca streamlet. Gorgova Lake is part of the Gorgova-Uzlina lowland area, located on the north-western side, south of the Sulina Branch [16].

Reed samples were collected from the area south of Dolosman Cape, between Razelm Lake and Golovita, part of the din Razelm-Sinoe Lagoon Complex of the Danube Delta, nearby Jurilovca village, Tulcea county, Romania.

### 2.2. Sample Processing and Analysis

Sampling of the reed batches was performed by the squares method. We used a square polypropylene frame with a 1 m^2^ surface in order to set the boundaries of the substrate from which we collected 16 shoots, including rhizomes [17]. After collection, the shoots were measured with a measuring tape and a caliper. Subsequently, the shoots were cut and stored in polyethylene bags at a −20 °C temperature onboard our ship. Those *Phragmites australis* samples were then washed in the laboratory with regular and then deionized water. The plants were cut and split into adventive roots, rhizomes, stems and leaves, in order to evaluate diversity of the shoot bioaccumulation [18]. The next step was oven drying at 180 °C for two hours, followed by shredding and mixing till homogeneity was reached, as sample homogeneity is often an underrated issue in environmental analysis [19,20,21,22].

After sorting the samples from the spots of interest in this study, we dried them in an oven at 50 °C in Petri dishes, which were subsequently double sealed with silicone adhesive to prevent air or humidity exchange with the environment. This is conducted for the following two reasons: (i) it has been proven that radon descendants’ contribution varies over time if an air flow exists, no matter how little—so the descendants’ contribution to the spectrum would be far from the radioactive equilibrium in the uranium–radium series; (ii) air exchange would also mean water intake for the samples, which leads on auto-attenuation coefficients increasing for the gamma rays, as well as underestimation of the specific activity (because it is defined as the number of disintegrations of a radioactive species per mass unit, wet samples would be heavier and would lead to a lower ratio). In order to reach the secular equilibrium in the U-Ra series, a waiting time of about 3 weeks is necessary after the sealing process, or some 6 half-lives of 3.82 days for ^222^Rn progenies to obtain 99% saturation; then, the generation rate for daughter nuclei equals the decay one. As Rn is a gas, sealing is an important issue, because any escape from the sample container leads to an equilibrium loss.

The measurement setup was a classic one. Samples were placed on top of the entrance window of a liquid-nitrogen-cooled Hyper-Pure Germanium detector (n-type, with a high efficiency even in the low-energy spectrum range) of 45% relative efficiency at 1332 keV and 2.1 keV at full-width at half maximum, high voltage source, amplifier, and multi-channel analyzer—all produced by ORTEC—and housed in a lead shield meant to reduce the radiation background. A typical acquisition time was around 24 h in order to obtain reasonable statistics. The spectra analysis was performed with the Interspec 1.0.12 and Maestro 32–7.0 software packages.

Each Petri dish has four weighing results marked on its top, as follows: dish weight (i); dish and net sample weight (ii); dish, net sample, and sealing adhesive weight after adhesive drying ~24 h (iii); and the same after three weeks of waiting and measurement sequence (iv). If the 3rd and 4th values differ, then the sealing process has failed. Several blank samples (empty Petri dishes identically sealed) were measured following the same procedure as the reed samples, previously sealed with the same amount of adhesive (a possible contaminant in certain cases) in order to correctly subtract the activity that did not originate directly from the samples and to better estimate the detection limits.

The a priori detection limit, L_D_, and the a posteriori critical limit, L_C_, as presented in the Section 3, were determined in accordance with the classic 1968 paper by Currie [23], which points out the need of treating this matter in terms of statistical tests. In 2004, De Geer refined this process [24], optimizing it for gamma-ray energy spectra. Following those definitions, the International Organization for Standardization recommended statistical hypothesis testing for characteristic limits determination, according to ISO 11929-1:2019, with an in-depth analysis for gamma radiation provided in 11929-3:2019 [25]. De Geer argued that the more information one has about the background in which the sample data have been acquisitioned, the more the statistical errors are diminished. As such, the critical and detection limits (in counts for the chosen measurement time) are as follows:

$$L_{C}=k\cdot \sqrt{1.25GB_{E}\omega_{E}\left(1+\frac{1}{m}\right)\;}\;;\;\;\;L_{D}=k^{2}+2L_{C}\;$$

where k is a constant related to the assumed risk translated into a fractile of the Gaussian distribution; G is the number of channels used per keV; $B_{E}$ is the measured background counts per energy unit; $\omega_{E}$ is the FWHM expressed in keV; and m is the number of trials (times the background has been measured). For most reasonable risks, with *k* < 5, *L_D_* is correctly approximated by 2*L_c_*. The statistical analysis was conducted using the XLSTAT 7.5.2 software [26].

## 3. Results

### 3.1. Specific Activities and Normalized Values

Following the spectral analysis, we calculated the specific activities (~per dry mass unit, in Bq·kg^−1^) for each sample, carefully subtracting the blank activities, not only the background from the individual spectra. In this respect, the detection limits were calculated for each nuclide-specific energy but this time also in Bq·kg^−1^ for the mean acquisition time (2 × 10^5^ s) and mean dry sample mass (16 g), providing the following levels in Table 1.

One interesting fact to point out is that the following results seem, sometimes, to be near to the detection limit or lower, which would be a contradiction. Additionally, according to De Geer, a signal near *L_D_* = 2*L_C_* would have an uncertainty of 30%, without the calibration source’s contribution. However, this situation can be simply explained, as follows: as opposed to the simple counts, calculating the detection limits per mass unit explicitly considers the detection efficiency, meaning the detection solid angle—so the background contributions are equivalent to a Marinelli-type measurement (almost *4π sr*) of the base line, while the sample only affects the entrance window. Consequently, the true detection limit for the geometry of interest is lower, which explains why the peak shape is decent even if the activity is at the apparent lower threshold. But this is another metrology topic currently being studied for such samples, namely, sometimes the blank container itself is less accountable than the surrounding background for the critical level analysis. Also, a low sample mass is a disadvantage; however, increasing the mass by increasing the volume (~sample height in this geometry) would also mean considering the self-attenuation and distance effects’ convolution, which result in lowering the overall detection efficiency. Each point of the resulting curves has its associated uncertainty, which is calculated according to the related budget, as summarized in the following equation:

$$u{\left(\Lambda s\right)}^{2}=u{\left(M\right)}^{2}+u{\left(U\right)}^{2}+u{\left(\varepsilon \right)}^{2}+u{\left(N\right)}^{2}+u{\left(I_{i}\right)}^{2}+u{\left(G\right)}^{2}+u{\left(El\right)}^{2}$$

Consequently, the uncertainty of the specific activity, $u\left(\Lambda s\right)$, is affected by the propagation of $u\left(M\right)$ on the mass, $u\left(U\right)$, or the non-uniformity component, $u\left(\varepsilon \right)$, on the detection efficiency, mainly due to the calibration source activity, where $u\left(N\right)$ is the counting statistics, $u\left(I_{i}\right)$ is the gamma yield, $u\left(G\right)$ is for the geometry, and $u\left(El\right)$ is for the electronics used. The samples were fixed on a specially designed ring on top of the detector. The analytical balance provided five significative digits, and the electronics were stable, and the yields are very precise. The uncertainty components, which need to be considered in this case, are those for the calibration source activity (10%), the source and sample uniformity (5%), and the counting statistics (following the case). Note that the error bars on the graphs are actually greater than the ones that need to be considered when comparing relative measurements of the samples. The 10% originating from the calibration source are systematically either toward an upper or lower value.

The results exhibit normal patterns, both for natural and anthropogenic contributions, as shown in Table 2. Regarding the nuclide classification, ^235^U (with a yield of 10.9%) has a contribution starting from ^223^Ra (yield 3.2%) at the 144 keV peak, which cannot be properly deconvoluted, and we do not know enough about the chemical composition in order to make further assumptions. The ^226^Ra and ^235^U also have a common peak at 186 keV; in fact, only 43% of the peak area belongs to ^235^U, if the radioactive equilibrium is valid in the U-Ra series (which should be correct, for the samples were sealed for more than three weeks before measurement, and the half-life of ^222^Rn is about 3.8 days; however, the results suggest, in some cases, that the quality of the sealant was questionable). As our main interest is to validate whether we can differentiate between the samples by using gamma spectral analysis, as long as no result departs from a normal order of magnitude, it is the relative differences among the samples that are of major importance. Of course, this does not withdraw the need for higher quality calibration sources in order to continue the study.

The sample numbering, type, and codes are as follows: Musura (leaves) ML2, Musura (leaves) ML1, Musura (stems) MS1, Musura (rhizome) MR1, Rosu-Rosulet (leaves) RRL, Rosu-Rosulet (stems) RRS, Rosu-Rosulet (rhizome) RRR, Rosu (rhizome) RR1, Jurilovca (leaves) JL1, Jurilovca (leaves) JL2, Rosu (stems) RS1, Rosu (leaves) RL1, Gorgova (stems) GS1, Gorgova (leaves) GL1, Gorgova (leaves) GL2, Jurilovca (rhizome) JR1, Gorgova (rhizome) GR1, and Jurilovca (stems) JS1.

**Figure 2:**
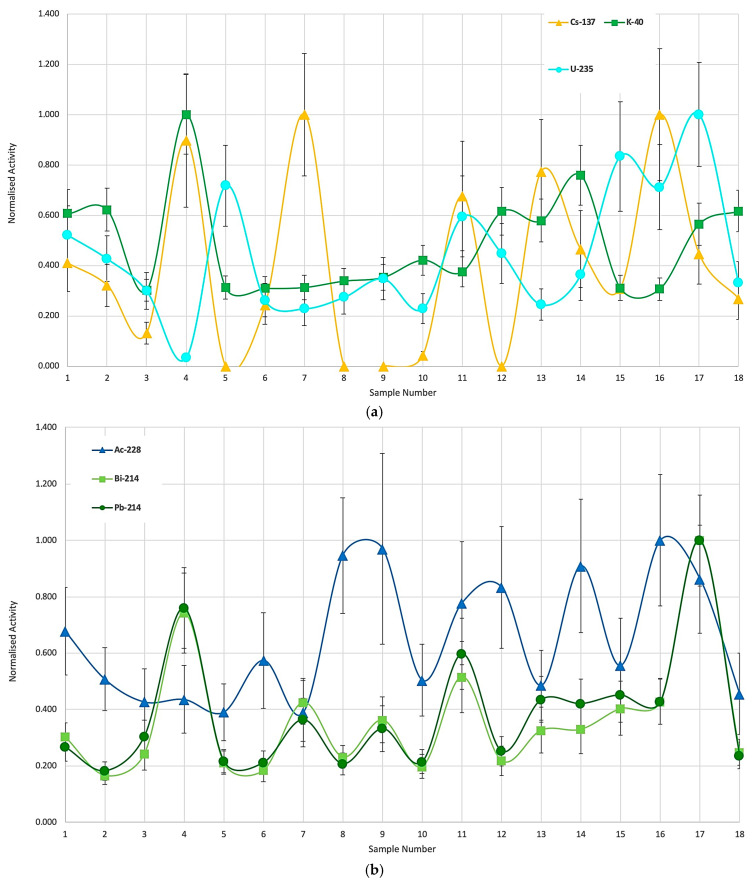
(**a**) Normalized specific activities for the uncorrelated radionuclides; (**b**) normalized specific activities for U-Ra series and actinium (thorium series).

In order to establish a common ground, we chose to compare those specific activities with each other by normalizing the values for each radionuclide to the highest one from the batch. We plotted separately K-40, which is a primordial, non-series, and natural radionuclide present in most lifeforms, depending on the spots and species; U-235, which is a standalone component for those datasets; and Cs-137, which represents the residual activity from the Chernobyl accident (1986) and earlier nuclear testing from back in the 1960s. We simply observed the individual contributions that are uncorrelated but within normal values (Figure 2a). Figure 2a,b represents the normalized activities but in a sequence depending on the sample part (rhizome, leaf, etc.), and then on the sampling area. This means points 1 to 5 correspond to rhizomes, 6 to 10 are stems, and 11 to 18 are leaves from the mentioned locations.

If we now chose to analyze the elements from the U-Ra series (such as Pb-214 and Bi-214) and the thorium series (such as Ac-228), common evolution patterns would arise for those from the same series and the differences from the others would become visible (Figure 2b).

It is evident that the dependence must further correlate with the soil/sediment analyses in order to calculate the specific transfer factors. Nevertheless, the purpose of this study was achieved, as we can distinguish between the samples and constituents with little effort.

Overall, the energy resolution and the high efficiency of the HPGe is accountable for the possibility of differentiating those samples of a few grams for a spectral acquisition time of about two days, which can be lowered to less than 24 h by adding extra shielding.

### 3.2. Neutron-Induced Emissions

**Figure 3:**
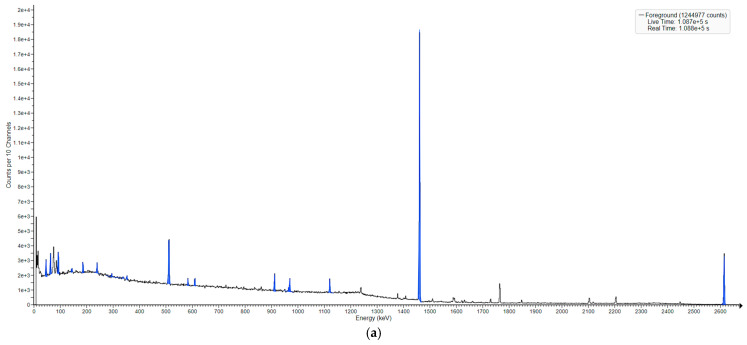

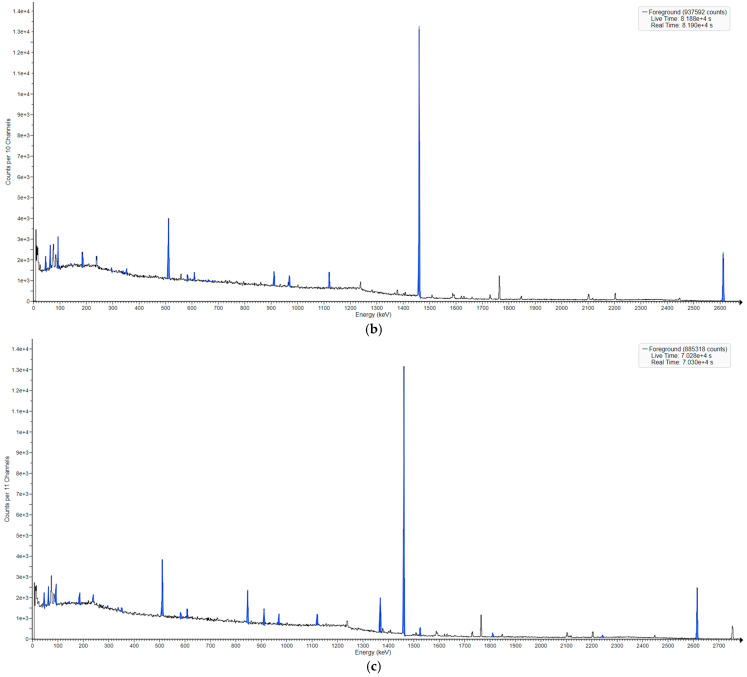

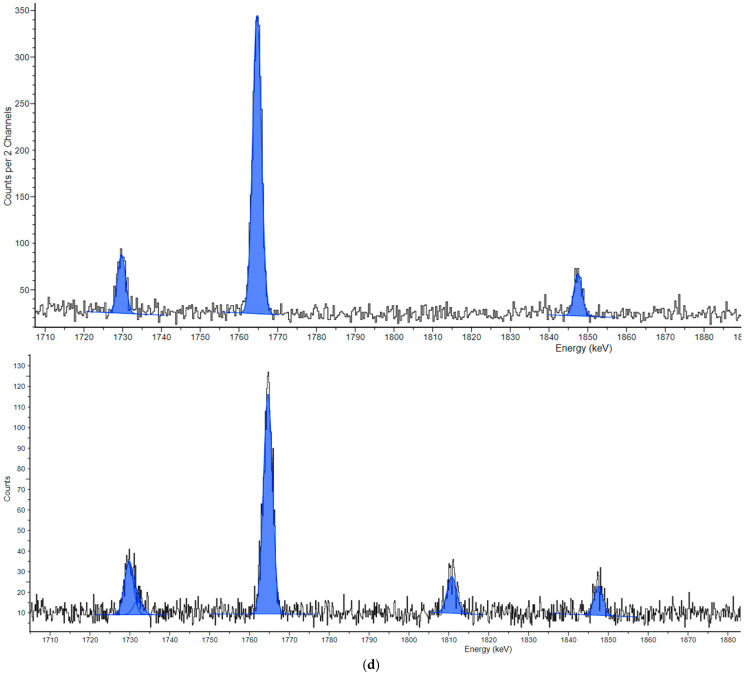
(**a**) Natural spectrum of the sample; (**b**) blank spectrum for the neutron-activated sample; (**c**) neutron-activated spectrum; (**d**) neutron-activated spectrum, with the 1710–1860 keV region, 1830 keV peak, and emerging convolution at 1730 keV (possibly from the Cl-36 contribution added by the natural Bi-214) visible. Given the spectrometer resolution of some at 3 keV for this energy, it is possible that this peak was also contributed to from the double escape of the second Na-24 peak (2754 keV).

In order to extend the analysis to the case of nuclides that were initially not radioactive or in an excited state (which allows for specific gamma emissions), we irradiated a sample (RRS) with a low-intensity thermal neutron flux (5–7 × 10^5^ n/s in 4π) and collected the resulting spectrum. After all of the background and blank activities were subtracted, we analyzed the differences between the natural and the irradiated spectrum results (Figure 3a–c).

Figure 3a,b display the regions of interest for the spectrum of the environmental sample and, respectively, the blank one, with the blank activities much lower. Figure 3c shows the new peaks, which do not occur naturally, at least not in such an environment.

Several neutron-induced emissions were identified, with the specific activity of a few tens of Bq/kg, among which are Mn-56 (846.78 and 1810.54 keV), Na-24 (1368.28 keV), and K-42 (1524.58 keV). A detailed view of the 1710–1860 keV region is provided in Figure 3d.

Neutron-induced nuclei did not exist in the natural sample, but their emissions prove their existence and, implicitly, the existence of their precursors. This is additional information on the sample’s composition, which can be evidenced by gamma measurements but only after the neutron’s capture. Even if the measurements were performed without a prior accurate characterization of the neutron source, as the irradiations and subsequent measurements were made in the exact same geometries, with same exposure and waiting time, the relative abundances are part of the signature of each sample from the batch. The accumulation of more elements can, therefore, be studied by analyzing the soil/sediment, plants, and the trophic chain.

### 3.3. Covariance and Clustering

**Figure 4:**
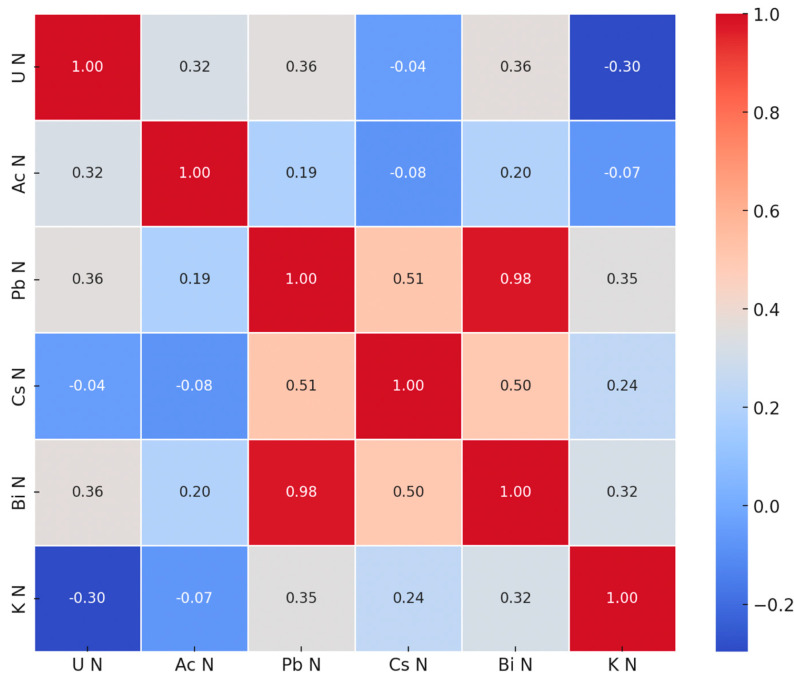
Correlation Matrix for the selected radionuclides.

**Figure 5:**
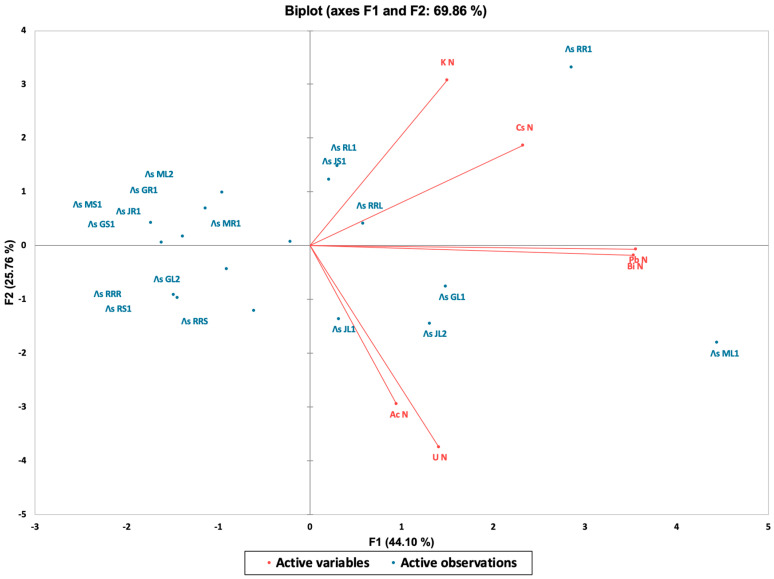
Biplot for the selected locations and radionuclides.

From the correlation analysis point of view, the batch exhibits strong discrepancies among the samples, which are linked to (at least) the sampling spot, period of sampling, specific part of the plant, which was prepared for the analysis. In other words, the correlation patterns were expected to be weak. Still, the results of the analysis speak for themselves (Table 3, Figure 4 and Figure 5). The dataset presents the relative normalized activities (Λs) of various radionuclides (U, Ac, Pb, K, etc.) measured in different plant tissues (leaves, stems, and rhizomes) from multiple sampling locations, as follows: Musura, Rosu-Rosulet, Rosu, Jurilovca, and Gorgova. The results highlight the distinct radionuclide distribution patterns, which are influenced by geographical location, plant type, and biological accumulation processes.

Strong positive correlations (such as between ^214^Pb and ^214^Bi, r = 0.98) show that the variation patterns are very similar, which is natural, especially for components of the same decay series. This strong correlation supports the measurements’ reliability and justifies their inclusion in the combined analysis (e.g., ratios and groupings). Moderate positive correlations (such as between Pb and Cs, r = 0.51) may reflect common accumulation pathways in certain plant tissues or a similar mobility in the environment (even if ^137^Cs is of anthropogenic origin). Moderate to weak positive correlations for other pairs (such as Pb and K, r = 0.35), indicate some degree of overlap in their geochemical or biological behaviors, while weak or negligible correlations exhibit independent plant uptake variability. The negative correlation between U and K reflects different uptake mechanisms or physiological regulatory processes in plants (e.g., K is biologically regulated, while U is not). The different correlation strengths also reflect the potential for using radionuclide ratios for environmental source tracing or ecological transfer factor studies.

This biplot presents the results of a principal component analysis (PCA), showing the relationships between samples (facility locations) and variables (radionuclides). The two axes represent the first two principal components. The first principal component (F1) explains 44% of the total variance in the data. This captures the direction of the maximum variability and reflects differences associated primarily with Pb, Bi, and Cs. The second principal component (F2) accounts for 26% of the variance and is orthogonal to (independent of) F1. This captures the variation driven primarily by K and, to a lesser extent, by Ac and U. The length of each red arrow indicates the contribution of the respective radionuclide to the variance. The direction and proximity between the arrows indicate the correlation strength. Pb and Bi are almost collinear, confirming the very strong positive correlation also observed in the matrix. U and Ac are closely aligned, indicating similar distribution patterns across samples, while Cs and K diverge from the rest, demonstrating independence in behavior. Each blue dot represents a plant sample from a specific location (e.g., ML1, JR1, and GL2) and their position in the biplot reflects the different radionuclide influences. For example, ML1 is strongly influenced by U and Ac—which are located in their direction—while RRS and RS1, which are located in negative quadrants F1 and F2, appear to be less influenced by any dominant radionuclide. This type of analysis can be used to identify the environmental or biological factors affecting radionuclide uptake and to explore the grouping or classification of sample types.

More than one element from the U-Ra radioactive series was used, for (i) these elements can form different chemical compounds—as allowed by their half-lives, especially those that are several hours in length—and these compounds may accumulate or diminish differently; (ii) the series includes a noble gas (Rn), for which there is a high chance of it escaping various parts of the plant with different kinetics, without even forming a compound, which is also a reason for further studying the concentration dynamics in the trophic chain.

As a side note, it is well known that a correlation of 0 does not imply independence, and a strong correlation may be related to coincidences, so this study needs to be extended. From the perspective of geographical areas, samples originating from the same region, such as Gorgova or Jurilovca, tend to exhibit higher covariances within their cluster, indicating a similar distribution pattern of radionuclides. Different areas (e.g., Musura and Gorgova) exhibit lower covariances, reflecting distinct local influences, such as soil type or vegetation. As for the biological significance, high covariances between leaves, stems, and roots within the same location suggest similar radionuclide accumulation patterns due to common biological or ecological processes.

The highest Cs-137 activities were observed in JS1 (Jurilovca stems) and JL2 (Jurilovca leaves, suggesting a significant retention of this anthropogenic radionuclide in those particular plant parts. Cs-137 was below the detection limit in several samples, which indicates either very low environmental availability or low uptake by these specific plant tissues. Cs-137 (Cs N) has a distinct distribution, reflecting its contaminant character; its distribution does not follow the pattern of natural radionuclides. There are strong correlations between radionuclides from the same decay series (Pb-214 and Bi-214). Potassium, a biologically essential element, exhibits significant variability but is relatively evenly distributed across samples, confirming its expected presence in plant tissues. The highest K-40 activity was found in RR1, while the lowest values appeared in JL2 (Jurilovca leaves, one-third of RR1). In terms of potential geographical influence, samples from Musura exhibited higher uranium contents, suggesting localized environmental factors influence their accumulation. Similarly, Jurilovca shows notable concentrations of Ac-228, indicating site-specific soil composition, while Cs-137 is linked to historical contamination. There is also a biological and ecological relevance for the radionuclide variability across plant tissues, highlighting distinct absorption, translocation, and retention capacities depending on the element’s chemical properties and plant metabolism.

## 4. Discussion

Interpretation of the experimental data points out that all measured radioactivity levels were within a normal range. K-40 is a naturally occurring primordial radionuclide that is found in all living beings, soils, sediments, etc. Cs-137 is an anthropogenic component that still bears witness to the Chernobyl event almost 40 years after the explosion. The U-235 levels are low, as normally expected. The Ac-228 values are also low, which is normal for the Th-232 series. Any small variation in the background, including the one for the blank samples results in a dramatic relative change for those series, of which elements are found in much smaller quantities in plants than in soils. For the U-Ra series, the values are normal and, at the same time, high enough to be detected together with reasonable differences from one sample to another, as pictured by the error bars, which are actually oversized (as stated in Section 3.1, they include a 10% systematic component that is irrelevant to activity ratios). We believe this will allow for cluster analyses in the nearby future.

Nevertheless, we must consider that specific activities can vary from one year to another due to transfer factor fluctuations but also due to various environmental parameters. In actuality, different values were obtained from the same spots while measuring samples during different seasons, stage of foliation, etc. Some aspects facilitate the analysis, such as the Ra/U ratio in soil, which was almost identical to the Ra/U in leaves, as shown in [27]; however, they cannot yet be generalized. In order to extend the study, the first thing is to perform an analysis of the associated soils and to validate that the cleaning and separation protocols did not allow the vegetation to carry any contaminants, in the sense that soil contains those radioelements in greater concentrations. Typical soil-to-plant transfer factors for the reeds were estimated to be about 0.1 [28].

This study should be continued along several directions. The first is the primary radio-elemental characterization of the biota components, including microelement analysis, which can be partially completed by neutron activation, although it is not mandatory. Second, contamination analysis brings valuable information on the anthropogenic components of the spectra, as shown here with the ^137^Cs example. Then, a covariance analysis can help cluster the samples in order to characterize and/or differentiate them but also to further study the potential contaminants’ migration. We believe it would be very useful to perform an in-depth investigation—first down to the sediment level and then upward along the trophic chain—in order to evaluate the radionuclides’ accumulation and transfer factors.

Although the values are not out of range for the biota, occasionally, a priori, unlikely ratios between radioactive descendants are observed. This suggests the spectra collection process may be sensible to small background variations, even if several trials of the background/blank were recorded. Analysis of such samples requires extra shielding, as the specific activities are lower than those of the soil/clay/sediment ones the laboratory has dealt with in the past. As shown, the radon activity in a container with an air layer above the sample was not uniform [29], displaying a surface effect in addition to a volume one, and the quantities must also be adapted in order to fill the Petri boxes.

The method development included adapting electronics for detection and shielding in order to obtain better energy resolutions and lower backgrounds, especially for in low-energy range, where hard X-rays interfere with the most sensitive part of the spectrum. The sampling procedure must be adapted for future work in order to provide larger quantities, most of all for vegetation—as the latter results in a much lower mass after the preparation protocols because of the high water content. Broadly, if a 10 cm thick lead shield, supplemented by an interior copper cover, of the spectrum of a typical 100 g dry sample is acquired with a 50% relative efficiency Hyper-Pure Germanium detector, then a 12 h time interval would be reasonable—which increases the capacity of small labs in terms of sample batches for analysis.

## 5. Conclusions

We proposed gamma-ray spectrometry as a method for biological sample characterization in order to evaluate the feasibility of an extended study in the Danube region, meant for characterizing the different matrices, from the sediment to the trophic chain components, and eventually for calculating the associated transfer factors. The results indicate that this is a suitable approach for non-destructive testing, which allows for differentiating between selected types, groups, and areas. Subsequent neutron activation enhances pattern recognition by adding valuable information from the gamma emissions following the beta decay processes post-neutron capture. Still, the sample is considered lost in this case, and it may become radioactive waste if the neutron flux is high.

In order to proceed with an in-depth study, we must perform a wider series of measurements for determining the specific activities related to all of the cited matrices periodically, for a few years. The method’s development must include refining peripheral electronics for detection, lowering the background, and, most of all, its fluctuations in the measurement setup, collecting enough samples of each type so the resulting dry quantities will be enough for a relatively short spectrum acquisition time. The relative or normalized activities determined are very well suited for feasibility evaluations, but for a regularity protocol for analysis from key spots [30], the efficiency calibration becomes, again, the most important issue for the laboratory—and even more important for inter-laboratory comparisons.

## Figures and Tables

**Table 1:** Detection limits for the gamma lines of interest.

Nuclide	^235^U	^228^Ac	^214^Pb	^137^Cs	^214^Bi	^40^K
Energy	144	338	352	662	1120	1461
D. L. (Bq·kg^−1^)	1.8	3.4	3.5	4.8	5.2	5.1

**Table 2:** Specific activities for the reed batch: Iγ (%) stands for the specific gamma yield, ε × 10^2^ for the experimental efficiency multiplied by 10^2^; Λs (X) for the X-specific activity in Bq/kg; u (X) for the associated uncertainty; “Norm” for the activities normalized to the highest in the batch; Dry (g) for the dry mass in grams; and LT (s) for the live time in seconds. All dead times were corrected by the Gedcke–Hale method.

E (keV)	144			338			352			662			1120			1461				
Iγ (%)	10.9	U-235		11.27	Ac-228		35.72	Pb-214		85.1	Cs-137		14.9	Bi-214		10.77	K-40			
ε × 10^2^	18.8			9.5			9.2			5.2			3.5			3.1				
No/Sample	Ls	u (U)	Norm	Ls	u (Ac)	Norm	Ls	u (Pb)	Norm	Ls	u (Cs)	Norm	Ls	u (Bi)	Norm	Ls	u (K)	Norm	Live (s)	Dry (g)
**1 Λs MR1**	5.48	0.22	0.52	8.61	0.23	0.68	3.79	0.19	0.27	0.82	0.27	0.41	4.56	0.17	0.30	238	0.16	0.61	3 × 10^5^	15.71
**2 Λs GR1**	4.49	0.21	0.43	6.45	0.22	0.51	2.58	0.18	0.18	0.65	0.26	0.32	2.50	0.16	0.17	244	0.14	0.62	3 × 10^5^	18.13
**3 Λs JR1**	3.16	0.25	0.30	5.42	0.27	0.43	4.32	0.19	0.31	0.26	0.32	0.13	3.65	0.17	0.24	118	0.15	0.30	2 × 10^5^	20.28
**4 Λs RR1**	0.35	0.29	0.03	5.53	0.28	0.44	10.78	0.19	0.76	1.80	0.30	0.90	11.16	0.17	0.74	392	0.16	1.00	2 × 10^5^	7.20
**5 Λs RRR**	7.54	0.22	0.72	4.96	0.26	0.39	3.07	0.19	0.22	0.00	0.26	0.00	3.18	0.17	0.21	123	0.15	0.31	2 × 10^5^	18.42
**6 Λs GS1**	2.75	0.25	0.26	7.30	0.30	0.57	3.00	0.19	0.21	0.49	0.31	0.24	2.78	0.17	0.18	121	0.15	0.31	2 × 10^5^	16.24
**7 Λs JS1**	2.41	0.29	0.23	4.95	0.31	0.39	5.16	0.21	0.36	2.01	0.24	1.00	6.40	0.19	0.43	123	0.16	0.31	96,600	16.07
**8 Λs RS1**	2.88	0.25	0.27	12.03	0.22	0.95	2.94	0.19	0.21	0.00	LD	0.00	3.48	0.17	0.23	133	0.15	0.34	2 × 10^5^	20.43
**9 Λs RRS**	3.65	0.24	0.35	12.32	0.35	0.97	4.71	0.25	0.33	0.00	0.19	0.00	5.46	0.20	0.36	139	0.15	0.35	1 × 10^5^	14.17
**10 Λs MS1**	2.41	0.26	0.23	6.40	0.25	0.50	3.04	0.20	0.21	0.09	0.35	0.04	2.98	0.18	0.20	165	0.14	0.42	2 × 10^5^	19.90
**11 Λs GL1**	6.25	0.27	0.59	9.88	0.28	0.78	8.47	0.21	0.60	1.36	0.32	0.68	7.76	0.20	0.52	147	0.16	0.38	80,200	17.15
**12 Λs GL2**	4.72	0.26	0.45	10.59	0.26	0.83	3.57	0.21	0.25	0.00	LD	0.00	3.28	0.19	0.22	241	0.15	0.61	1 × 10^5^	19.48
**13 Λs RL1**	2.58	0.26	0.24	6.17	0.25	0.49	6.18	0.19	0.44	1.55	0.27	0.77	4.91	0.18	0.33	227	0.15	0.58	2 × 10^5^	19.00
**14 Λs RRL**	3.82	0.28	0.36	11.54	0.26	0.91	5.97	0.21	0.42	0.93	0.33	0.46	4.96	0.19	0.33	298	0.16	0.76	87,300	19.32
**15 Λs JL1**	8.77	0.26	0.83	7.06	0.30	0.56	6.41	0.21	0.45	0.62	LD	0.31	6.07	0.20	0.40	122	0.16	0.31	85,900	12.75
**16 Λs JL2**	7.48	0.24	0.71	12.70	0.23	1.00	6.06	0.19	0.43	2.01	0.26	1.00	6.44	0.17	0.43	120	0.15	0.31	2 × 10^5^	10.94
**17 Λs ML1**	10.52	0.21	1.00	10.95	0.22	0.86	14.17	0.16	1.00	0.89	0.27	0.45	15.03	0.15	1.00	221	0.15	0.56	4 × 10^5^	5.48
**18 Λs ML2**	3.50	0.25	0.33	5.78	0.32	0.46	3.33	0.20	0.24	0.54	0.31	0.27	3.72	0.18	0.25	242	0.13	0.62	2 × 10^5^	18.64

**Table 3:** Distribution moments of the normalized specific activities of the batch.

Isotope	Minimum	Maximum	Mean	Std. Dev
U-235	0.033	1.000	0.437	0.248
Ac-228	0.390	1.000	0.650	0.221
Pb-214	0.182	1.000	0.383	0.217
Bi-214	0.166	1.000	0.363	0.214
K-40	0.302	1.000	0.484	0.198
Cs-137	0.000	1.000	0.388	0.350

## Data Availability

The raw data that led to the results presented in this work are available upon request, including the individual spectra of the samples and blank trials.
